# Fanconi anemia complementation group C gene (FANCC) association with hereditary and sporadic renal tumors

**DOI:** 10.1093/oncolo/oyag012

**Published:** 2026-01-20

**Authors:** Devashish Desai, Rebecca A Sager, Michael Basin, Joseph M Jacob, Gloria Joan Morris, Philippe E Spiess, Roger Li, Liang Cheng, Andrea Necchi, Ashish M Kamat, Petros Grivas, Dean Pavlick, Hanan Goldberg, Mehdi Mollapour, Douglas Lin, Jeffrey S Ross, Gennady Bratslavsky, Alina Basnet, Michael A Daneshvar

**Affiliations:** Division of Hematology and Oncology, Department of Hematology and Medical Oncology, SUNY Upstate Medical University, Syracuse, NY, USA; Department of Urology, SUNY Upstate Medical University, Syracuse, NY, USA; Department of Urology, SUNY Upstate Medical University, Syracuse, NY, USA; Department of Urology, SUNY Upstate Medical University, Syracuse, NY, USA; Division of Hematology and Oncology, Department of Hematology and Medical Oncology, SUNY Upstate Medical University, Syracuse, NY, USA; Department of Urology, H. Lee Moffitt Cancer Center and Research Institute, Tampa, FL, USA; Department of Genitourinary Oncology, H. Lee Moffitt Cancer Center and Research Institute, Tampa, FL, USA; Department of Pathology, Warren Alpert Medical School of Brown University, Providence, RI, USA; Vita-Salute San Raffaele University, Milan, Italy; The University of Texas MD Anderson Cancer Center, Houton, TX, USA; Department of Medicine, Division of Hematology/Oncology, University of Washington, Fred Hutchinson Cancer Center, Seattle, WA, USA; Foundation Medicine, Inc, Cambridge, MA, USA; Department of Urology, SUNY Upstate Medical University, Syracuse, NY, USA; Department of Urology, SUNY Upstate Medical University, Syracuse, NY, USA; Foundation Medicine, Inc, Cambridge, MA, USA; Department of Urology, SUNY Upstate Medical University, Syracuse, NY, USA; Foundation Medicine, Inc, Cambridge, MA, USA; Department of Urology, SUNY Upstate Medical University, Syracuse, NY, USA; Division of Hematology and Oncology, Department of Hematology and Medical Oncology, SUNY Upstate Medical University, Syracuse, NY, USA; Department of Urology, University of California, Irvine Health, Orange, CA, USA

**Keywords:** kidney cancer, FANCC, hereditary kidney cancer, comprehensive genomic profiling

## Abstract

**Background:**

Inactivating genomic alterations (GA) of the *FANCC* gene are associated with genomic instability, DNA cross-linking, and homologous DNA repair deficiency. Here, we evaluated the incidence of *FANCC* GA in renal tumors (RT).

**Methods:**

A total of 463 546 clinically advanced cancer (CAC) cases underwent hybrid capture-based comprehensive genomic profiling using the FDA-approved F1CDx assay to detect all classes of GA. Microsatellite instability (MSI) status, tumor mutational burden (TMB), genomic loss of heterozygosity, prediction of germline status, and genomic signature were determined with algorithm-based analysis.

**Results:**

Clinically advanced cancers (0.43% of 1993) featured FANCC GA; 27 of these *FANCC*-mutated tumors (20 males, mean age 57) were RT (0.35% of 7668 RT). The primary tumor was sequenced in 9 cases and a metastatic site in 18 (5 lymph nodes, 4 soft tissues, 3 brains, 2 livers, 1 each lung, adrenal, eye, bone). Only 1 of 25 tested FANCC-mutated RT was MSI-high; 4 cases (15%) featured TMB ≥10 mutations/Mb. The genomic signature could be assessed in 5 cases: 4 were MMR-deficient. The *FANCC* mutations included inactivating short variant mutations in 24 cases (10 nonsense, 10 frameshift, 2 non-frame, and 2 splice-site mutations) and 3 truncating rearrangements (FANCC: SUSD3, FANCC: FANCC, and FANCC: C20orf24). Interestingly, 14 (52%) of the *FANCC*-mutated RT were predicted to be germline.

**Conclusions:**

Somatic and germline mutations in *FANCC* occur in an exceedingly small subset of clinically advanced RT but at a similar rate to other cancers, and the genomic landscape does not appear to be different from RT with wild-type *FANCC*. Germline testing is warranted, as we see a high frequency of germline FANCC mutations.

**Patient Summary:**

Our study highlights the rate of FANCC mutation in kidney cancer, which may be a therapeutic target and awaits further assessment and drug development. Also, it shows that FANCC mutations are more germline, requiring further genetic testing.

Implications for Practice The current trend in further utilization of next-generation sequencing to analyze the tumor genetics and its application in finding targets for newer therapies has led us to do a study on highlighting the incidence of Fanconi anemia complementation group C (FANCC) mutation in cancers, especially renal tumors (RT). We found that the FANCC mutation is present in a low number of patients, and its genomic profile is similar to that of FANCC wild-type RTs. Also, RTs with mutated FANCC were predominantly having germline mutations and hence require further genetic testing. FANCC could be a target to induce synthetic lethality, though currently in trials.

## Introduction

**Figure 1.**
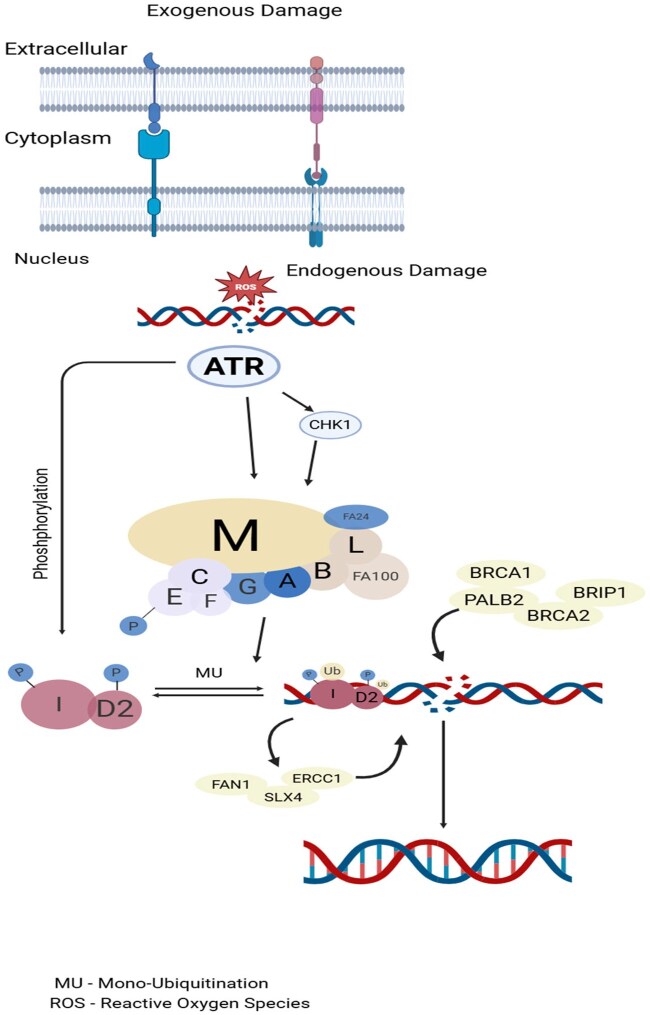
The schematic elucidation of the FA pathway mechanism used during DNA repair. In response to exogenous and/or endogenous damage, 8 FA genes (FANCA, FANCB, FANCC, FANCE, FANCF, FANCG, FANCL, and FANCM) assemble into the FA core complex, which functions as a nuclear E3 ubiquitin ligase complex, to monoubiquitinate the I-D heterodimer. The monoubiquitinated I-D heterodimer is localized to the damaged chromatin and interacted with DNA-repair proteins and other FA proteins (FANCD1, FANCDN, FANCJ, and FANCS) in the FA pathway, to conduct the repair process through homologous recombination. After the damage was repaired, monoubiquitin was removed from the I-D complex by a de-ubiquitylation enzyme, Ubiquitin Specific Peptidase 1, to “turn off” the network.^2^

Fanconi anemia (FA) is a rare autosomal or X-linked recessive genetic disease spectrum causing a broad spectrum of clinical features of variable penetrance, mainly progressive bone marrow failure (depending on the affected gene), congenital defects, and cancer predisposition.^1^^,^^2^ A total of 22 genes have been described as FA genes: *FANCA, FANCB, FANCC, FANCD1/BRCA2, FANCD2, FANCE, FANCF, FANCG/XRCC9, FANCI, FANCJ/BRIP1, FANCL/PHF9, FANCM, FANCN/PALB2, FANCO/RAD51C, FANCP/SLX4, FANCQ/ERCC4, FANCR/RAD51, FANCS/BRCA1, FANCT/UBE2T, FANCU/XRCC2, FANCV/REV7*, and *FANCW/RFWD3.*^3^ As seen in Figure 1, the FA pathway (also known as FA-BRCA pathway) is a fundamental DNA repair pathway in the S phase of cell cycle that recognizes DNA damage and aids in its repair, especially for DNA interstrand crosslink (shown in Figure 1).^4^ Fanconi anemia genes play a crucial role in tumor suppression or maintenance of genomic stability among general population, as FA pathway inactivation has been seen in various tumor types in non-FA patients.^5^

Fanconi anemia complementation group C gene (FANCC) is the first identified FA gene, accounting for about 14% of FA cases.^6^ Genomic alterations (GA) in FANCC (“FANCC mutations”) are almost exclusively inactivating and cause genomic instability, DNA cross-linking, and homologous DNA damage response deficiency. Fanconi anemia complementation group C mutations are most frequently associated with colorectal, prostate, lung, and breast cancers, with germline mutations linked to familial breast and ovarian cancers.^7–10^ There is a paucity of data for FANCC mutations in renal tumors (RT), and they account for less than 1.0% of FANCC-mutated tumors in the Foundation Medicine database. Fanconi anemia complementation group C mutations in RT are not currently linked to any hereditary renal cancer predisposition syndromes. In this study, we evaluated the incidence of FANCC mutations and the genomics of FANCC mutations in RT

## Methods

### Sample and setting

Approval for this study, including a waiver of informed consent and a HIPAA waiver of authorization, was obtained from the Western Institutional Review Board (Protocol No. 20152817). DNA was extracted from 463 546 clinically advanced cancers (CAC), which included 7688 cases of RT and underwent hybrid capture-based comprehensive genomic profiling (CGP) using the FDA-approved F1CDx assay to detect all classes of GA.^11–15^

### Analysis

The central laboratory (Foundation Medicine) used for CGP is Clinical Laboratory Improvement Amendments-certified and accredited by the College of American Pathologists. A minimum of 50 ng of DNA was extracted from cases with a minimum of 20% tumor nuclei. After library preparation, adaptor-ligation-based hybrid capture was performed for all coding exons of 324 (version 3) cancer-related genes plus select introns of 28 (version 3) genes frequently rearranged in cancer. The Illumina HiSeq was used for DNA sequencing to a mean exon coverage depth of >600X. Microsatellite instability (MSI) status was determined on 95 loci. Tumor mutational burden (TMB) was determined using 0.9-1.1 Mb of sequenced DNA. The DAKO 22C3 CDx assay was used to determine PD-L1 expression using 5-micron tissue sections. Following the CDx assay guidelines, a tumor proportion score (TPS) was determined for each sample stained with the DAKO 22C3 CDx assay. TPS = (positive tumor cells/total tumor cell) × 100. TPS of 0% was defined as negative, low-level staining was defined as 1%-49% TPS, and high-level staining was defined as ≥50% TPS. Genomic loss of heterozygosity (gLOH), prediction of germline status, genomic ancestry, and genomic signature were determined by algorithm-based analysis.^11–15^ The prediction of germline status in this study used a computational method for predicting somatic or germline status from the deep sequencing results and is based on genome-wide copy number modeling: somatic-germline zygosity (SGZ) utilizes a model of genome-wide copy number to account for alterations like amplifications and deletions in the tumor genome. The SGZ model predicts germline status based on the VAF, copy number profile, and tumor purity.^12^

### Statistical analyses

Differences in sample medians were assessed using the unpaired Mann–Whitney–Wilcoxon test. Differences among categorical variables were assessed using the chi-square test with Yates correction. Statistical tests were 2-sided and used a significance threshold of *P* < 0.05. Reported *P*-values were not adjusted for multiple testing.

## Results

**Figure 2.**
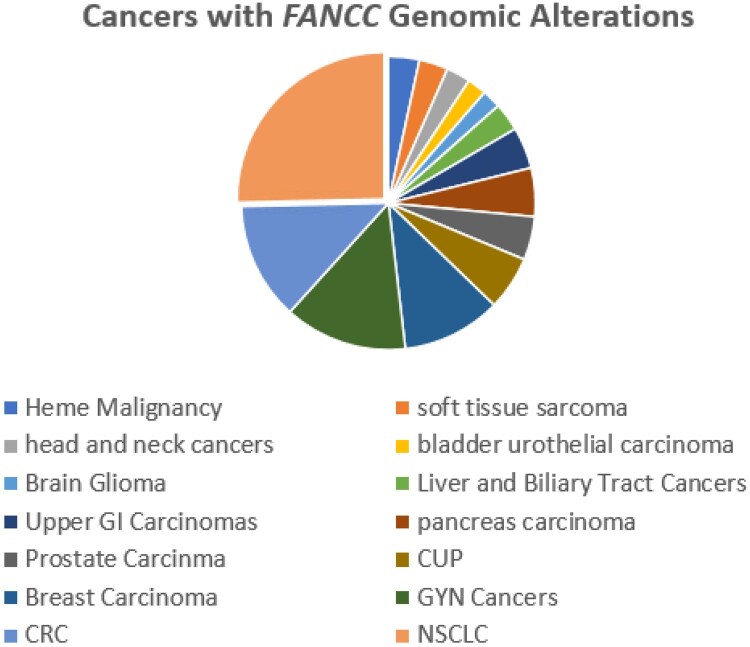
Cancers with FANCC Genomic Alterations. RCC cases account for only 0.4% of FANCC-mutated cancers in the Foundation Medicine database.

When the entire database of tumors was assessed, FANCC mutations were most commonly seen in non-small cell lung carcinoma (∼25%), followed by colorectal cancers, gynecological cancers, breast cancers, cancer of unknown primary, prostate carcinoma, and so forth (Figure 2).

A total of 1993 (0.4%) cases of the CACs in the Foundation Medicine database were identified as having FANCC mutations. Twenty-seven of these FANCC-mutated tumors were RT (0.35% of 7668 RT), which were more frequently identified in males (74.07% [*n* = 20]) and had a mean age of 57 years. On reviewing the pathology of the FANCC mutated RTs, the majority were found to be clear cell (48.2% [*n* = 13]), followed by sarcomatoid (11.1% [*n* = 3]), urothelial (11.1% [*n* = 3]), chromophobe (11.1% [*n* = 3]), squamous cell (7.4% [*n* = 2]), medullary (7.4% [*n* = 2]), and Wilm’s tumor (3.7% [*n* = 1]). The primary tumor was sequenced in 9 cases and a metastatic site in 18 (5 lymph nodes, 4 soft tissues, 3 brains, 2 livers, 1 each lung, adrenal, eye, bone).

As seen in Table 1, the median TMB in the FANCC-mutated RT was 2.5 mutations/Mb. Four (14.8%) cases had ≥10 mutations/Mb. For the FANCC wild-type KT, the median TMB was also 2.5 mutations/Mb, and 1.9% of these tumors featured a TMB of ≥10 mutations/Mb. The mean gLOH was 7.5%. Twenty-five cases of FANCC RT were tested for MSI status, of which 1 (4%) had MSI-high. For the FANCC wild-type KT, only 0.2% were MSI-high; 2 of 5 (40%) FANCC RT tested for PD-L1 were low positive, and 1 (20%) was high positive. For the FANCC wild-type RT group, low PD-L1 expression was found in 25.6%, and high PD-L1 expression was found in 10.4% of cases.

**Table 1.** Comparison of major genomic alterations and immunotherapy biomarkers in FANCC-mutated and FANCC wild-type renal cell carcinomas.

	FANCC-mutated RCC	FANCC wild-type RCC
**VHL**	60.9%	56.1%
PBRM1	26.1%	31,2%
**TP53**	26.1%	17.7%
**SETD2**	21.7%	21.5%
**PTEN**	13.0%	9.9%
TMB in mutations/Mb median	2.5	2.5
**TMB ≥10 mutations/Mb**	14.8%	1.9%
**MSI High**	4.0	0.2%
**PD-L1 low expression (1-49% TPS)**	40.0%	25.6%
**PD-L1 high expression (≥50% TPS)**	20.0%	10.4%

**Figure 3.**
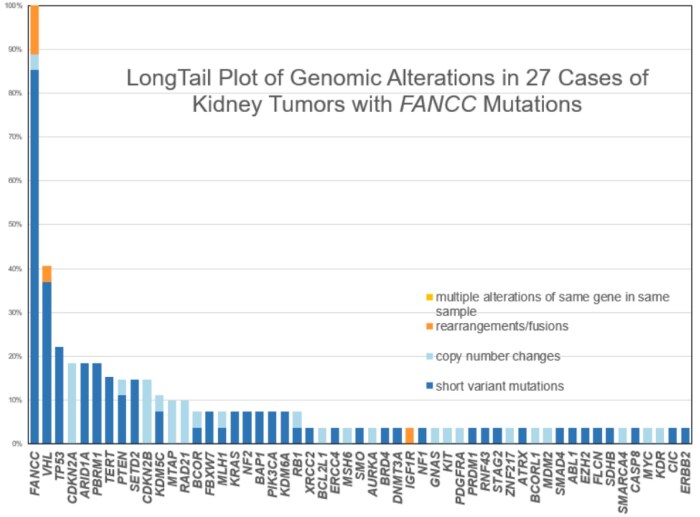
The long-tail plot of all classes of Genomic Alterations in 27 cases of FANCC RT. The frequency of mutations is shown on the Y axis, and the names of the most frequently mutated genes are shown on the X axis.

**Figure 4.**
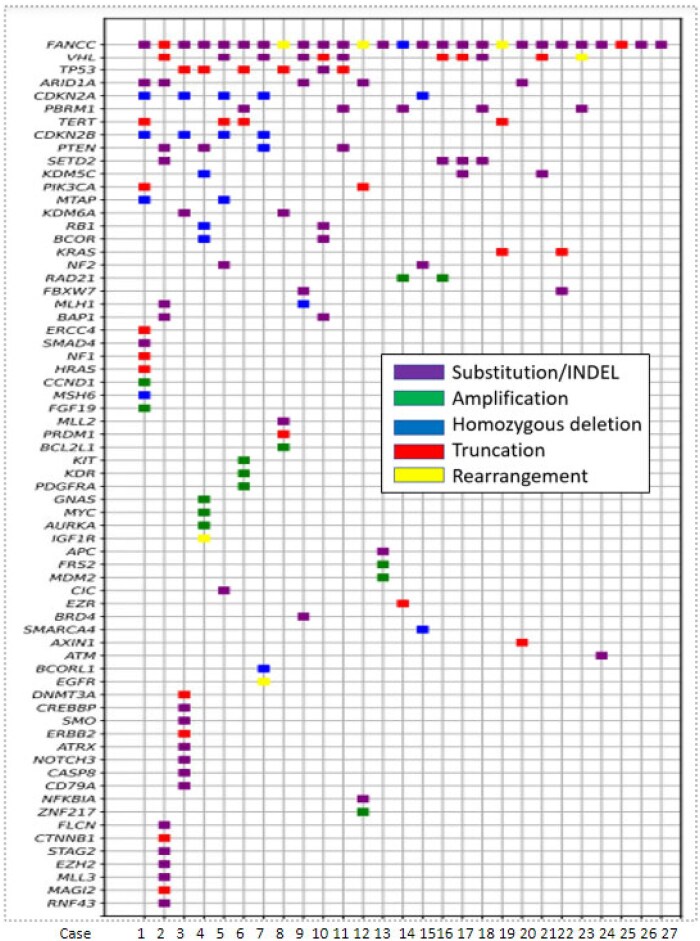
The tile plot of all classes of genomic alterations in the 27 cases of FANCC-mutated KT.

**Figure 5.**
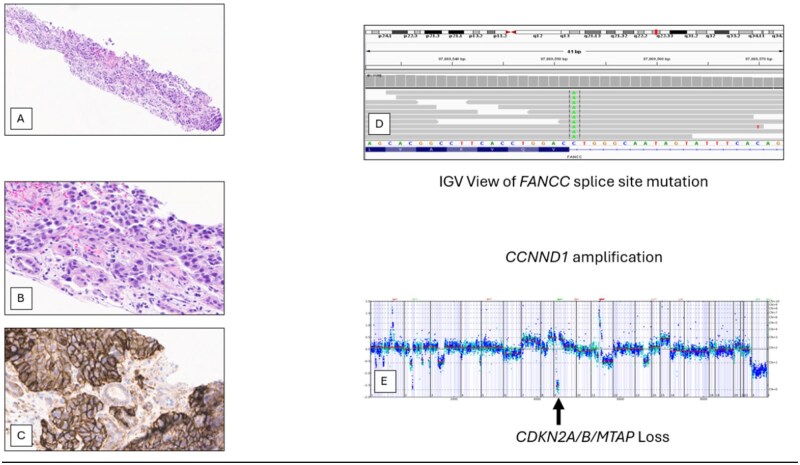
High-grade urothelial carcinoma of the right renal pelvis in an 80-year-old man with a prior history of prostate cancer. On IHC staining (A, B), this tumor was positive for CK7, p63 PAX8, and GATA3 and negative for PSA, RCC, and CK20. PD-L1 IHC staining (C) was high (100% TPS) positive. On comprehensive genomic profiling, this tumor was MS-stable and had a high TMB of 23 mutations/Mb. The tumor featured a FANCC splice site 1330-1G>T mutation (D) predicted to be somatic only along with additional short variant mutations in ARID1A S645*, ERCC4 A583T, NF1 T482A, PIK3CA N345T, HRAS G12D, SMAD4 Q388*, SMAD4 S504R, and TERT promoter -124C>T. The copy number plot (E) revealed homozygous deletion of CDKN2A/B and MTAP with amplifications of CCND1 and FGF19. Implications for treatment selection for this patient may have included immuno-oncology drugs based on the high TMB and PD-L1 expression. In addition, this patient would have potentially been eligible for entry into a clinical trial featuring either a PRMT5 or MTAT2a inhibitor based on the MTAP loss.

**Figure 6.**
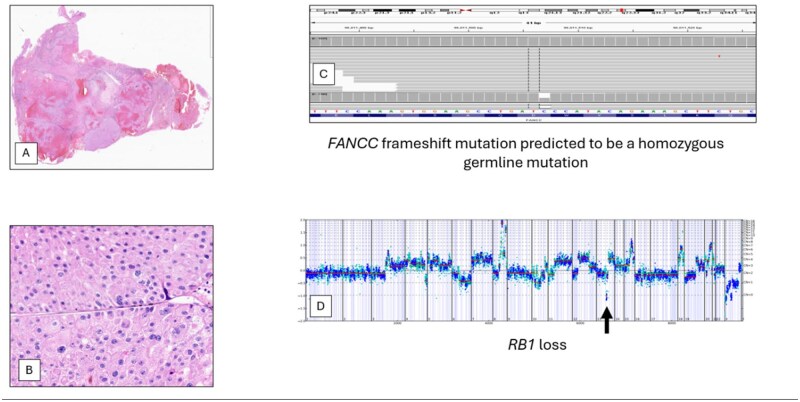
Partial resection of a brain metastasis in a 75-year-old man with a history of chromophobe RCC (A, B). This patient had undergone a radical nephrectomy 3 years previously. On comprehensive genomic profiling, this MS stable tumor had a low TMB of 1 mutation/Mb. This tumor had a frameshift FANCC D23fs*23 mutation at 60% allele frequency predicted to be a germline mutation (C). Short variant mutations in PTEN Y336fs*8 and TP53 E258G were identified along with an IGF1R(NM_000875)-PIAS1(NM_016166) fusion. The copy number plot revealed losses in KDM5C, BCOR, and RB1 along with amplifications of MYC, AURKA, and GNAS (D). Stable disease was achieved for this patient with a combination of cabozantinib and nivolumab.

On evaluating all classes of FANCC GAs in the RT cases, the alterations were predominantly inactivating short variant mutations. Of those 27 FANCC-mutated RT, the majority were nonsense mutations (*n* = 12), frameshift mutations (*n* = 11), followed by non-frame mutations (*n* = 2), and splice-site (*n* = 2) mutations. Of the 27 FANCC-mutated RT, 11.1% (*n* = 3) had truncating rearrangements (ANCC: SUSD3, FANCC: FANCC, FANCC: C20orf24). Fourteen (52%) of the FANCC-mutated RT were predicted to be germline by the SGZ protocol. Genomic ancestry evaluation revealed 21 EUR (European), 4 AFR (African), and 2 AMR (admixed American) patients. Important frequently mutated genes in the FANCC-mutated RT and FANCC wild-type RT are shown in Table 1 and included mutations in of VHL 60.9% vs 56.1%, PBRM1 (26.1% vs 31.2%), TP53 (26.1% vs 17.7%), SETD2 (21.7% vs 21.5%), and PTEN (13.3% vs 9.9%) (Figures 3 and 4). Case examples of FANCC-mutated RT are shown in Figures 5 and 6.

## Discussion

The FANCC gene is a component of DNA damage-induced E3 monoubiquitin ligase, an essential and integral part of the FA-BRCA pathway in damaged DNA repair.^16–18^ Cells deficient in FANCC will have defects in double-stranded DNA repair by homologous recombination (HR). In such cases, cells will try to repair the damage with error-prone non-HR mechanisms, culminating in genomic instability.^19^ Poly-(ADP-ribose) polymerase (PARP) is a signal transducer protein that binds to damaged single-stranded DNA and synthesizes poly (ADP-ribose) on the targeted breaks in the domain of DNA and PARP itself, which allows the recruitment of other repair proteins to complete the repair process.^19^ Loss of PARP can lead to increased repairs by the FA-BRCA pathway, which would be lethal in FA-BRCA pathway defects, including FANCC GA. This phenomenon was scrutinized and led to the development and use of PARP inhibitors (PARPi) in BRCA1/BRCA2-mutated cancers.^20^ FANCC GA also leads to HR deficiency, and causes “BRCAness phenotype” and may result in tumor sensitivity to PARPi.^21–23^ There have been various phase I/II umbrella trials where the efficacy of PARPi has been noted in FANCC-mutated cancers, especially in breast cancer, lung cancer, ovarian cancer, and in other advanced solid malignancies.^24–30^

The Foundation Medicine cohort used for this study revealed that only 0.43% of the advanced cancers had FANCC mutations, with these cases most frequently identified in non-small cell lung cancer, followed by colorectal carcinoma, and only 1.36% seen in RT. On reviewing the AACR Genie Portal, FANCC GA occurs in about 0.97% of all cancers, with the most commonly occurring in colorectal cancers, followed by lung adenocarcinoma, endometrial adenocarcinoma, breast invasive ductal carcinoma, and prostate adenocarcinoma. It is seen in about 1.1% of clear cell renal cell carcinoma.^31^ The frequency of FANCC mutations in the clinically advanced clear cell RCC in the Foundation Medicine database is 0.3%. These higher frequencies of FANCC mutations in the AACR Gene Portal, which includes both early and late-stage disease, compared with the Foundation Medicine advanced cancers only database suggests the possible favorable impact of FANCC mutation status on tumor stage at diagnosis and clinical outcome that calls for further investigation. The relatively small number of FANCC-mutated RT in this study precludes the ability to compare the patterns of genomic alterations based on histologic subtype.

As seen in Table 1, the genomic profile of the FANCC-mutated RT is not very different from wild-type FANCC RT.^32^ The small differences seen in the table for mutations in VHL, PBRM1, TP53, SETD2, and PTEN were not statistically significant.

An important finding in this study is the prediction of germline status for more than half (52%) of the FANCC mutations that were identified. These are predicted to be germline mutations that clearly warrant formal germline testing for those patients, and based on the germline test results, these results can subsequently guide the counselling for family members.

### Future perspectives for FANCC-mutated cancers

Along with FA-BRCA pathways, there are other parallel pathways for DNA repair. In periods of FA-BRCA pathway deficiency, cells tend to lean on or compensate for the DNA repair mechanism using various other pathways, and inhibiting those will cause cell damage. The FANCC gene, being one of these FA-BRCA pathways, utilization of PARPi may ultimately be warranted pending ongoing clinical trial results. It is likely that umbrella trials testing clinical responsiveness to PARP inhibitors in all types of solid tumors will be conducted, incorporating HR genes and HR signatures. Other targets for synthetic lethality are being developed as well. With siRNA screening, it is seen that genes such as ATM, TREX2, PARP1, PLK1, UBE2B, and many more, if inhibited, can also lead to synthetic lethality. In an in vitro setting, it has been seen that ATM inhibition in both human fibroblasts and murine embryonic fibroblasts with FA pathway deficiency shows synthetic lethality.^33^ Similarly, a rationale was utilized by inhibiting CHK1. In FA-BRCA pathway deficiency, CHK1, which controls G2/m checkpoint, is hyperactivated, leading to the accumulation of cells in the G2 phase, allowing them to undergo repair of interstrand crosslink damage. It is hypothesized that inhibiting this checkpoint, by inhibiting CHK1, will aid in synthetic lethality, which was proven by Chen et al.^34^

In conclusion, the FANCC mutation is identified in a small subset of RT. FANCC-mutated RT do not appear to be significantly different from FANCC wild-type KT in other genomic and biomarker characteristics and thus may be eligible to participate in upcoming umbrella studies for HR-deficient cancers. In addition, more than 50% of FANCC-mutated RT were predicted to feature germline FANCC mutations, warranting further genetic testing and counselling. Finally, the lack of clinical annotation and clinical outcome data in this study is a major limitation that prevents a more expanded discussion of the results.

## Author contributions

Devashish Desai (Writing—original draft), Rebecca Sager (Writing—review & editing), Michael Basin (Supervision, Writing—review & editing), Joseph Jacob (Supervision, Writing—review & editing), Gloria Morris (Supervision, Writing—review & editing), Philippe Spiess (Supervision, Writing—review & editing), Roger Li (Supervision, Writing—review & editing), Liang Cheng (Supervision, Writing—review & editing), Andrea Necchi (Supervision, Writing—review & editing), Ashish Kamat (Supervision, Writing—review & editing), Petros Grivas (Supervision, Writing—review & editing), Dean Pavlick (Methodology, Software), Hanan Goldberg (Supervision, Writing—review & editing), Mehdi Mollapour (Supervision, Writing—review & editing), Douglas Lin (Methodology, Software), Jeffrey Ross (Conceptualization, Supervision, Writing—review & editing), Gennady Bratslavsky (Supervision), and Alina Basnet (Supervision, Writing—review & editing), Michael Daneshvar (Supervision, Writing—review & editing)

## Data Availability

Data available on request.
